# Open Dialogue in Spain: an initial survey of knowledge and perspectives

**DOI:** 10.3389/fpsyg.2023.1166919

**Published:** 2023-08-10

**Authors:** Silvia Parrabera-García, Casilda Oujo-Fernández, María-Jesús Lirola, Adolfo J. Cangas, Jordi Marfá-Vallverdú, Martín Correa-Urquiza, Enric Garcia-Torrents

**Affiliations:** ^1^Hospital Universitario Príncipe de Asturias, Madrid, Spain; ^2^Galicia Mental Health System, Galicia, Spain; ^3^Department Psychology, University of Almería, Almería, Spain; ^4^Catalonia Mental Health System, Catalonia, Spain; ^5^Medical Anthropology Research Center, Universitat Rovira i Virgili, Tarragona, Spain

**Keywords:** Open Dialogue, implementation, mental health, new perspectives in healthcare, psychotherapy training

## Abstract

In Spain, the introduction of the Open Dialogue framework is relatively recent. This study takes a closer look at Open Dialogue training, interest and research in this region. To this end, a survey has been conducted through a convenience sample of professionals, people with their own experiences in mental health, family members, relatives, university professors and students. The results showed that a significant number of participants had no training in OD, and their exposure to relevant literature and congress attendance was limited. Amongst the different profiles, professionals reported the highest level of training. These findings highlight the urgent need for further research and training initiatives to improve the understanding and application of the OD framework in Spain. Efforts should be directed towards broadening the knowledge base, increasing access to training programmes and fostering interest amongst different stakeholders. By addressing these gaps, the implementation and use of OD can be expanded to meet the growing demand and interest in this approach in the Spanish context.

## 1. Introduction

In recent years, there has been a growing interest and a gradual introduction of the Open Dialogue (hereinafter, OD) framework as an alternative treatment approach in Spain. Originating in Western Lapland in the 1980s, OD has demonstrated significant success in reducing the incidence of psychosis, achieving a remarkable decrease from 33 to 3 cases per 100,000 inhabitants over the course of a decade (Seikkula and Arnkil, 2016). The effectiveness of this intervention is primarily due to the basic principles underlying the OD framework, which can be summarised as follows (Seikkula et al., 2006, 2011): First, the provision of immediate help, within a 24-h timeframe following a request for help. In addition, networking plays a key role in OD, including family members and community members who can contribute to the well-being of the person seeking support. In addition, OD offers considerable flexibility in treatment, allowing adaptations to be made to meet the specific needs of each individual. In addition, the collaborative nature of OD is exemplified by professionals working together as a team, usually consisting of two to three members. Long-term continuity of care is emphasised, with follow-up and treatment extending over a minimum period of two to three years. In addition, OD encourages the cultivation of tolerance for uncertainty, discouraging hasty decisions such as urgent hospitalisation or excessive reliance on medication. Finally, OD meetings are characterised by the principles of dialogue, ensuring active participation and equal voice for all members involved.

This OD approach bears remarkable similarities to mutual support groups, as highlighted by Chmielowska et al. (2022) and Lorenz-Artz et al. (2023). Its adoption extends beyond Spain, as evidenced by its use in several countries, as reported by Buus et al. (2021) and Mosse et al. (2023). Although the adoption of OD in Spain is relatively recent, significant progress has been made. In 2016, it was first used as a tool in the Mental Health Centre of Badalona (CSM Badalona 2), specifically to support recovery processes, following a pilot experience (Vallverdú et al., 2019). Subsequently, in 2017, the health authorities of the Community of Madrid approved and promoted the use of OD as a therapeutic framework and organisational system in the Early Attention Unit for Psychosis (UAT) of the Príncipe de Asturias University Hospital in Madrid. However, the continuity of OD implementation in both centres faces challenges. In Badalona, the retirement of the person in charge, Dr. Jordi Marfà, has affected the continuity of the service, whilst in Madrid, changes in the team and the sick leave of the promoter, Silvia Parrabera, have resulted in a limited number of cases being treated from an OD perspective.

In particular, OD practises have also emerged outside the public system. Some associations, groups and collectives, such as *Laporvenir*, have developed their approaches based on the OD framework. Several of the founding members of Laporvenir were previously part of the UAT team at the Hospital Universitario Príncipe de Asturias, together with other institutions (see Parrabera, 2017). Although the association is facing economic difficulties, it continues its activities (more information can be found on its website: https://laporvenir.org/).

The emergence of new evidence highlighting the need to reassess the development of mental health systems, programmes and services is not unique to Spain. It is a trend that can be observed in Spain as well as in other European countries (Martín López-Andrade, 2015; Correa-Urquiza, 2017; Desviat, 2020; Huertas, 2020; Fernández Liria, 2022). These calls for reassessment highlight the importance of exploring alternative approaches, such as OD, to meet the evolving challenges and demands in the field of mental health.

The detrimental consequences of psychiatric diagnoses (Hyman, 2010; Colina et al., 2021), the increasing violation of rights within mental health services (Muñoz Escandell, 2021), and the limitations of a vertical, unidirectional model of care with limited emphasis on dialogue (Martínez-Hernáez, 2000) all highlight the need for transformative change. Desviat (2020) points out that the psychiatric reform of the 1980s was not a revolution, but a carefully negotiated transition involving psychiatric authorities from the dictatorship era who held influential academic and clinical positions, this reform did not fundamentally change the existing dynamics. However, the current context underlines the urgent need for change that recognises the inherent complexity of mental health problems and the associated social distress (Kleinman and Kleinman, 2000). Desviat (2020) advocates a ‘renewed clinic’ that includes essential elements such as continuity of care, therapeutic accompaniment, crisis intervention, home hospitalisation and the formation of transdisciplinary teams.

In this context of renewal, OD emerges as a transformative approach to the provision of care and support, with a strong emphasis on cultivating relationships based on complicity, proximity and compassion. It advocates dialogue and the deconstruction of hierarchical approaches to treatment, actively involving additional actors such as family members, neighbours or friends in the processes of therapeutic recovery. OD is based on the fundamental premise that mental health care is a collaborative and multidimensional endeavour that prioritises the reconstruction of relational aspects and the life trajectories of individuals, rather than focusing solely on pathology (Fernández-Villardón et al., 2022).

The implementation of OD in Spain is characterised by regional differences. In some cases, professionals have incorporated OD into their individual practises or integrated it with other existing methods, such as multifamily group therapy (Sala, 2020; Sempere and Fuenzalida, 2021; Oujo-Fernández et al., 2023; Sala, 2023) or contextual therapies, including acceptance and commitment therapy. In the latter case, however, the integration is more theoretical than based on specific training in OD (Díaz-Garrido et al., 2023). In addition, the involvement of experts with lived experience is a common practise within the care team.

The growing momentum of OD is in line with the need for a paradigm shift in the approach to mental health care, not only in Spain but also globally in the Western world (Hyman, 2010; Martín López-Andrade, 2015; Correa-Urquiza, 2017). OD has emerged as a response to the limitations and chronic effects of conventional biomedical treatments. It also reflects the dissatisfaction expressed by individuals with lived experience and professionals themselves, who feel constrained by distressing institutional dynamics that prioritise harm reduction through the use of psychotropic drugs and prevent the coherent implementation of their principles in meeting people’s needs (Tsou, 2007; Hyman, 2010; Beresford et al., 2016).

In Spain, people with lived experience of mental health services report the need for social change at all levels of the health system to include more supportive practises, fairness and respect for biocultural diversity (Hyman, 2010; Correa-Urquiza et al., 2020). This highlights the need for a cultural shift towards a more democratic and humane approach that recognises mental suffering as a multifaceted reality that requires careful consideration of its inherent complexity. Furthermore, changes in the working conditions of healthcare professionals are crucial to enable a more psychosocial approach and effective coordination that avoids isolating individuals from their unique circumstances (Tizón, 2013, 2014; Seikkula and Arnkil, 2016; Seikkula and Arnkil, 2019).

In response to the changing landscape of mental health care in Spain, OD is gaining relevance as an approach that meets the expectations of both professionals and individuals experiencing mental distress. Its value lies in its potential to reorganise the mental health system and transform professional practise through its open and flexible methodology. In addition, OD has the versatility to be applied in other community organisations. The growing interest in OD was exemplified by the recent 26th International Congress of the OD Network for the Treatment of Psychosis, held in Spain in 2022, which marked an important milestone for the OD approach.

Regarding training, which is fairly recent, first offered in 2020 as a University Expert Course in OD: Fundamentals were developed at the Universitat Ramon Llull in Barcelona (20 ECTS, 500 h), led by Dr. Berta Vall Castelló. The course had a first edition, but did not continue perhaps due to the economic cost, as it was a face-to-face course with several international speakers. An online course of 150 h of duration was launched in 2022 at the University of Almeria, which covered all its initial places (45) and is now preparing its reedition and the possibility of continuing this first promotion with a Level II (trainer of trainers). This course is co-directed by Jaakko Seikkula himself.

Thus, there have been some attempts to promote OD training and practise in Spain, but with various difficulties. What has not been carried out so far is a study on the opinion of people who had contact with OD in order to better understand their assessment of what this training entails and the changes it can represent in mental health in Spain. This study aimed to fill this gap.

## 2. Materials and methods

### 2.1. Participants

The target population was a convenient sample of professionals, people with their own experiences in mental health, family members, relatives, university professors and students. The recruitment was made by disseminating the link to the survey carried out in google forms, sharing the link in different instant messaging groups and through social networks. The inclusion criteria were to belong to one of the five groups mentioned above, regardless of age or previous OD experience.

### 2.2. Instruments

For data collection, a survey was designed collecting socio-demographic data (age, gender, level of studies, current occupation) and, subsequently, different questions related to:

1. Degree of knowledge of OD
2. OD training received
3. Possible implementation of OD
4. Participation in OD

The survey can be consulted in the Supplementary material. Likewise, when answering the questionnaire, participants could select one of the following profiles, leading to a series of questions about their experience with OD:

- People with own experiences in mental health
- Close friends / Environments
- Mental health professionals
- Public mental health system managers / associations with experience in OD
- University lecturers
- University students

To end with, an open question to the participants was included, namely “Finally, we welcome your thoughts, ideas, comments, observations, opinions on OD in Spain.”

### 2.3. Procedure

The aforementioned survey was designed and published using Google Form. A brief summary on the nature of the study was included at the beginning of the survey explaining it was anonymous and completely voluntary, and that participants could stop completing the questionnaire at any time. In addition, a contact point with the researcher team was provided. The questionnaire took between 15–20 min to complete. The study was approved by the Bioethics Committee of the University of Almeria (UALBIO2021/013).

Convenience sampling was used to gather participants, sending the form to the researchers’ databases containing people who had been in contact with OD, either because they had been involved in a clinical process based on OD or because they had undergone training. In order to avoid double entries for the online questionnaire response, the restriction of sending only one response per registered email was used. It was equally disseminated on social networks and WhatsApp groups to which the research members had access. No follow-up was carried out for those who did not respond to the survey.

### 2.4. Analysis

Descriptive statistics were calculated for the population. Subsequently, the responses obtained for each of the proposed questions on knowledge of OD were analyzed, obtaining frequency and distribution statistics for each of these variables. The different analyses were carried out using the SPSS statistical package in version 25.

## 3. Results

### 3.1. Descriptive statistics

A total of 214 people (147 women and 67 men) participated in the present study. The ages of the participants ranged from 18 years to over 70 years of age (55% of the population is between 30 and 49 years of age). Descriptive data on the participants were according to the four age brackets proposed as possible responses, we found from oldest to youngest with 4 participants aged 70 and over; with 29 people aged 60 to 70; a total of 33 subjects aged 50 to 59; another 61 people aged 40 to 49; with 60 participants aged 30 to 39; and, finally, 35 respondents aged 18 to 29. In terms of educational level, 87.4% had completed university studies.

Table 1 shows the distribution of the sample in terms of the six profiles collected, and whether they have received training in OD (40.19%) or not (59.81%).

**Table 1:** Descriptive statistics on profiles.

Profiles	*N*	NT	NT %	WT	WT %
People with their own experiences	29	20	68.97	9	31.03
Close friends / Environments	30	25	83.33	5	16.67
MH professionals	140	78	55.71	62	44.29
Public MH System Managers / Associations	5	1	20	4	80
University teachers	5	1	20	4	80
University students	5	3	60	2	40
Totals	214	128	59.81	86	40.19

Use: NT, No training in OD; WT, With training in OD.

Table 2 shows the time spent on training in OD, according to the profile of the participants. In this case, it can be seen that the profiles of public health managers and university professors have the highest rates of training in OD (80%) and, in third place, the profile of health professionals with 44.29% of these having undertaken some type of training in OD. However, this training has been limited in time, as only 16 people out of the total sample received more than 100 h of training (i.e., 12% of the total number of those who received some type of training).

**Table 2:** Training time in OD in hours.

	0 h.	1–5 h.	5–30 h.	30–100 h.	100–300 h.	+ 300 h.	% child h.
People with their own experiences	20	3	3	3	0	0	31.03
Close friends / Environments	25	2	0	2	0	1	16.67
MH professionals	78	10	22	15	13	2	44.29
Public MH System Managers / Associations	1	0	2	2	0	0	80
University teachers	1	0	3	1	0	0	80
University students	3	0	1	1	0	0	40
Total	128	15	31	24	13	3	40.19

Table 3 shows the distribution by country of origin of the training received by the participants. It can be seen that the majority was in Spain (almost 90%), with 4 people having received training in Argentina or Uruguay, 3 in England and 1 in Mexico.

**Table 3:** Origin of the training received.

	*N*	%	% Accumulated
Spain	68	89.47	89.47
Argentina-Uruguay	4	5.26	94.73
England	3	3.95	98.68
Mexico	1	1.32	100
Total	76	100	-

Table 4 includes frequency statistics of the participants who received some kind of training in OD, the year in which they first heard about OD, also the readings they have done on OD, attendance at talks or conferences on OD, and, finally, whether they have participated in any group or association to use OD as a resource for support. As can be seen, practically all the people begin to know about OD from 2020 onwards, except for mental health professionals, who indicate 2018. The number of readings on OD is also higher in professionals (7.73) and lower in the rest of people, as well as attendance at talks or organisation of sessions on OD, which is once again much higher in mental health professionals.

**Table 4:** Knowledge and application of OD.

	Year of knowledge OD	Numbers of OD readings	Numbers attendance talks	Organisation of sessions in OD
People with their own experiences	2020 (*n* = 7) 2022 (*n* = 2)	4,2	3,6	1
Close friends / Environments	2020 (*n* = 5)	5	0	0
MH professionals	2018 (*n* = 41) 2020 (*n* = 14) 2022 (*n* = 7)	7,73	6,19	7
Public MH System Managers / Associations	2020 (*n* = 4)	1,3	2,3	0
University teachers	2021 (*n* = 4)	2,7	3,5	0
University students	2020 (*n* = 2)	1,3	2	0

With regard to the perception of the need for a change in the care model of the public mental health system, results showed that 85% of those surveyed are in favour of changes, compared to 1.4% who think that changes are not necessary, and 13.6% who do not know/do not answer.

### 3.2. Qualitative analysis of the reflections on the OD in Spain

Using a method of syntactic analysis of the responses to the question “Finally, we would like to thank you for your thoughts, ideas, comments, observations, opinions on the OD in Spain,” four main blocks or central themes were identified: (1) Benefits of OD, (2) Lack of training, (3) Need for research, and/or, (4) Need for changes in the public mental health system.

With regard to the first category, we find that the participants highlight the importance of being able to rely on this methodology in treatment, emphasising the need for humanisation, normalisation of the experiences and the monitoring of cases in a much closer and less traumatic way, both for the user and for the people or family members around them. As textual evidence recovered from the responses, the following can be cited:

*“Very interesting type of therapy. The user and the family feel well supported. The results are evident for everyone”* (Woman, retired, 111).

*“I think it is a very interesting new treatment conceptualisation especially in psychotic patients that can reduce psychiatric admissions, as well as better link patients”* (Female, health, 127).

As for the second category, reference is made to the lack of training in OD in Spain. The possibilities and potential of OD are commented on, but also the need for courses or specialised training in the participants’ work centres to facilitate its implementation within the public mental health system. In this sense, the following reflections were made:

*“It is difficult to find where to get training”* (Woman, health, 28).

*“It seems that more is beginning to be known and disseminated, but knowledge is still very scarce, and there are many female workers within the MH system who would like to work with a different methodology that is more coherent with their values, and that does not put them in uncomfortable situations that take away agency from the people they care for”* (Woman, health worker, 45).

Thirdly, there is a need for more research in OD for its dissemination and the expansion of knowledge about the impact that this methodology of care for mental health users could have on the course of crises and care for both patients and families during their recovery process.

*“Publicity campaigns and good marketing are needed to make it known, as well as research studies that accredit and endorse it in a generalised way”* (Mujer, sanitaria, 52).

*“Need to publish studies to promote its application in public settings”* (Woman, health, 72).

The fourth and last category contemplates the need for changes in the public mental health system, for the inclusion of new approaches and ways of treating and monitoring people with serious mental disorders. It is essential to make changes and promote new health practises in order to really achieve greater progress within the public mental health systems and to evolve towards new horizons with more optimistic perspectives.

*“I don't really know how well established it is, its current situation, but I feel that a change in the way we look at mental health is necessary. Our society is governed by a rigid scheme based on scientific knowledge that generates stigmas, labels … closing off possibilities, not allowing us to see what person we have in front of us. OD and its dissemination can help to change this view”* (Woman, health, 35).

*“The public health system is still far from being able to incorporate models based on collaborative and dialogic practises”* (Woman, health, 123).

## 4. Discussion

The aim of this study was to evaluate the assessment and knowledge in Spain, a country where the first dialogic practises have recently been implemented, being important the holding for the first time in Spain the 26th International Congress of the OD Network for the Treatment of Psychosis in 2022.

The data obtained indicate that in the sample consulted there is a strong interest in a change in mental health, where OD can be a promising alternative, albeit there is still little knowledge about this framework. Thus, a significant percentage of respondents (almost 60%) indicate not having received any training on this approach, with the majority of those who have had some kind of training having received less than 100 h. This probably relates to the fact that there are few training possibilities in Spain, where there was only an initial course in 2020 at the Universitat Ramón Llul en Barcelona, which was not followed up, and another one recently at the University of Almeria. Nevertheless, the latter has sold out and is currently being considered for reissue, as well as the extension of the training to a Level II (trainer of trainers), thus that the impact it can have on mental health in Spain is likely to begin to be felt soon. This aspect, the training, seems to be key for OD to really bring about a real transformation in mental health in Spain.

The number of readings on this approach was low. The available readings in Spanish on this topic are also scarce, where there are hardly any articles or book chapters, concentrated in the last five years (Parrabera, 2018, 2019; Vallverdú et al., 2019, 2020; Abad and Toledano 2022; Oujo-Fernández et al., 2023; Parrabera-Garcia and Chico, 2023), with the exception of one work (Abad et al., 2015). Similarly, the majority of respondents indicate that they have only heard about this topic three years ago (since 2020). Only healthcare professionals are the ones who have heard about OD a little earlier (since 2018) and have read more or attended talks or conferences on this topic.

There is a high level of interest in the institutional recognition of OD as a legitimate practise and perspective for addressing mental health in the consulted sample; it is also essential to start applying to other community organisations in order to generate a social transformation and a cultural change (Seikkula and Arnkill, 2019). In this sense, although there are seminars and small training proposals, there is a clear need to broaden and deepen the creation of systematized and organised training. In this sense, 85% of respondents expressed the need for a paradigm shift in Mental Health, which can be linked to the mandate of the “United Nations Convention on the Rights of Persons with Disabilities” (2006) and the successive reports of the UN Special Rapporteurs in defence of these rights. The OD can be deduced as one of the possible methodologies for the materialisation of the transition (World Health Organization, 2021).

These results are similarly observed in the qualitative evaluation, where participants highlight the benefits of OD, the absence of training, the need for research and the importance of changing the public health system. Thus, it is true that there is hardly any research carried out in Spain, beyond describing some experiences of initial practise sites (Minondo Romeroa et al., 2022), but no funded projects in this area have been found, nor active participation in other international studies, such as HopenDialogue (https://www.hopendialogue.net/).

It is necessary to develop also more local research that measures and analyses its effectiveness, taking into account the socio-cultural particularities of the country’s context and territory. It is therefore necessary to analyse local casuistry in the implementation of the OD in order to produce evidence that allows us to evaluate the development and implementation of the model. Depending on these results, the possibility of endorsing the OD framework as a treatment option within the public MH system, and as specialised training in universities and scientific societies, could be considered.

In addition, the critical situation of the biomedical model in the field of mental health, promotes the urgency of new paradigms, practises and methodologies that accommodate the necessary transformations to generate a model attentive to the inherent complexity of the phenomenon of mental suffering. It is in this context that, for professionals, users and family members, OD appears as a possibility that, although it does not take into account the multiple dimensions related to this field, it is understood as a cornerstone on the road to the necessary transformations. It is an internationally legitimised possibility (World Health Organization, 2022) whose value lies, in turn, in the capacity at source to measure and analyse the impact of the model. In other words, the capacity of those who started with the OD to produce evidence of the results of its implementation is one of the key aspects of its international legitimacy.

Nevertheless, this study has some limitations such as the small sample size, particularly amongst some sectors. As a future line, it is considered important to repeat the study in the coming years, to see if knowledge of this approach improves and if this framework becomes established in clinical practise.

## 5. Conclusion

The present study analyses the knowledge and appreciation of OD in Spain by a sample of participants who have mostly had contact with this approach., where the most of the participants highlight the need for change that can be brought about by adopting the OD framework in our country, but also identifies a series of shortcomings, such as the need for more research, the few readings consulted by most of the participants and also a need for more training, particularly long-term training, which could make it easier for people interested in the subject to become involved in this change. It should be borne in mind that the introduction in Spain is still very recent, for example, the two most important training events that have taken place so far, both in 2022, are very recent, such as the 26th International Congress of the Open Dialogue Network and the first promotion of the University Expert in Open Dialogue in Mental Health at University of Almería has just finished, therefore it will be important to continue evaluating its implementation and their repercussions in the coming years, as well as new training, clinical and research experiences that will be carried out.

## Data availbility statement

The original contributions presented in the study are included in the article/Supplementary material, further inquiries can be directed to the corresponding author.

## Ethics statement

The studies involving humans were approved by Bioethics Committee of the University of Almeria (UALBIO2021/013). The studies were conducted in accordance with the local legislation and institutional requirements. The participants provided their written informed consent to participate in this study.

## Author contributions

SP-G, CO-F, AC and EG-T: conceptualization and visualization. AC: supervision. EG-T and M-JL: methodology and Validation. AC, SP-G, and EG-T: resources. SP-G, CO-F, M-JL, AC, JM-V, MC-U, and EG-T: investigation and writing—original draft preparation. AC, M-JL and MC-U: writing—review and editing. All authors read and agreed to the published version of the manuscript.

## Funding

This work has been financed with the support of the Spanish Ministry of Education by a University Professor Training Scholarship (FPU19/00028) granted to ET and Junta de Andalucía by a postdoctoral contract (DOC_01290) possible by its Directorate-General for Research and Knowledge Transfer awarded to M-JL.

## Conflict of interest

The authors declare that the research was conducted in the absence of any commercial or financial relationships that could be construed as a potential conflict of interest.

## Publisher’s note

All claims expressed in this article are solely those of the authors and do not necessarily represent those of their affiliated organizations, or those of the publisher, the editors and the reviewers. Any product that may be evaluated in this article, or claim that may be made by its manufacturer, is not guaranteed or endorsed by the publisher.
