# Supplementary material for: Open Dialogue in Spain: an initial survey of knowledge and perspectives
Source: Front Psychol. 2023 Aug 10;14:1166919. doi: 10.3389/fpsyg.2023.1166919 (PMC10448387; doi:10.3389/fpsyg.2023.1166919)
Supplement: Supplementary file 1 [file Data_Sheet_1.PDF]

ASSESSMENT OF THE CURRENT SITUATION OF OPEN DIALOGUE IN SPAIN

This questionnaire is part of the data collection of a research study, carried out by professionals from the Hospital Príncipe de Asturias in Madrid, the Universitat Rovira i Virgili, the University of Almería, the Ramón Llull University, the Galician Health Service and the Mental Health Centre 2 in Badalona, on the implementation of the **Open Dialogue** (hereinafter, ODF) **framework** in Spain.

It is **anonymous**, so we ask you to be as honest as possible when completing it. We also ask you to do it calmly, thinking and meditating on each answer.

Your participation in this study is **voluntary**. The estimated response time is around 10-15 minutes.

If you would like to request additional information, please contact us at the following email address: **dialogoabiertospain@gmail.com**

Thank you very much for your collaboration.

*Mandatory

1. If you would like to receive the report resulting from this survey please provide your email address below, otherwise leave this box blank:

2. Age *

Check only one category

Less of 18
Between 18-29
30-39
40-49
50-59
60-70
Over 70

3. Gender *

Check only one category

Male Female Other

4. Level of studies finished until he moment *

Check only one category

Primary school
High school Degree
Half - v o c a t i o n a l training
Higher vocational training
Degree University

5. Occupation current *

Check only one category

Student
Professional sanitary
Other

6. If you have answered Other in the ask former specify which:

7. Place of residence *

Check only a category

Álava (País Vasco, ES)
Albacete (Castilla- La Mancha, ES)
Alicante (Comunidad Valenciana, ES)
Almería (Andalucía, ES)
Asturias (Principado de Asturias, IS)
Ávila (Castilla y León, ES)
Badajoz (Extremadura, ES)
Barcelona (Cataluña, ES)
Burgos (Castilla y León, ES)
Cáceres (Extremadura, ES)
Cádiz (Andalucía, ES)
Cantabria (Cantabria, ES)
Castellón (Comunidad Valenciana, ES)
Ciudad Real (Castilla-La Mancha, ES)
Córdoba (Andalucía, ES)
Cuenca (Castilla-La Mancha, ES)
Gerona (Cataluña, ES)
Granada (Andalucía, IS)
Guadalajara (Castilla-La Mancha, ES)
Guipúzcoa (País Vasco, ES)
Huelva (Andalucía, ES)
Huesca (Aragón, ES)
Islas Baleares (Islas Baleares, ES)
Jaén (Andalucía, ES)

La Coruña (Galicia, ES)
La Rioja (La Rioja, ES)
Las Palmas (Islas Canarias, ES)
León (Castilla y León, ES)
Lérida (Cataluña, ES)
Lugo (Galicia, ES)
Madrid (Comunidad de Madrid, ES)
Málaga (Andalucía, ES)
Murcia (Región de Murcia, ES)
Navarra (Comunidad de Navarra, ES)
Orense (Galicia, ES)
Palencia (Castilla y León, ES)
Pontevedra (Galicia, ES)
Salamanca (Castilla y León, ES)
Santa Cruz de Tenerife (Islas Canarias, ES)
Segovia (Castilla y León, ES)
Sevilla (Andalucía, ES)
Soria (Castilla y León, ES)
Tarragona (Cataluña, ES)
Teruel (Aragón, ES)
Toledo (Castilla-La Mancha, ES)
Valencia (Comunidad Valenciana, ES)
Valladolid (Castila y León, ES)
Vizcaya (País Vasco ES)
Zamora (Castilla y León, ES)
Saragossa (Aragón, ES)

8. Where develop your occupation? *

Check only one category

Health system
Private sector
Subsidised centre
Social services
Association
Mutual aid group
Education
Other

9. If you have answered Other in the ask former specify which

____

1O. Have you received training in OD? *

Check only one category

Yes *Skip to question 11*
No *Skip to question 34*

ABOUT OPEN DIALOGUE (OD)

11. How long ago was your most intense training?*

Check only one category

1 year
2 years
3 years
4 years
5 years
It has been more than 5 years

11. How much time have you accumulated in OD training?*

Check only one category

Between 1 and 5 hours
Between 5 and 30 hours
Between 30 and 100 hours
Between 100 and 300 hours
More than 300 hours

12. Have you received training in Spain either in the foreign? *

Check only one category

Spain England Finland
Argentina-Uruguay
Other

13. If you have answered Other in the ask former specify which

---

14. What kind of training in the foreign? *

Check only one category

I have not received training abroad
Basic training for OD facilitators
Full 3/4 year training in OD
International Certification Training in Dialogic Practice
Basic OD training supported by peers and social networks
OD training of trainers programme
Other

15. If you have answered Other in the ask former specify which

---

17. What kind of training in Spain? *

Check only one category

In Spain I have not received training
University training
Continuous training
From an association
From a collective

16. Have you been part of some process of OD? *

Check only one category

Yes
No *Skip to question 34*

PARTICIPATION IN OPEN DIALOGUE

17. If you have formed part of some process indicate which

____

18. Have you been part of any OD process?*

Check only one category

Yes
No

19. If you have been part of any OD processes, please indicate which processs

____

20. Have you facilitated OD processes?*

Check only one category

Yes

No

21. If you have facilitated OD processes, please specify where

____

22. Have you organized any collective or association to use the OD as a resource of accompaniment?*

Check only one category

Yes

No

23. If you have organized some collective either association for use the OD as accompanying resource; specify which one:

24. Have you ever been part of a team using OD in Mental Health?*

Check only one category

Yes
No

25. Have you implemented the framework in any device belonging to the mental health network?*

Check only one category.

Yes
No

26. If you have implemented the frame in some device belonging to the Mental Health Network; specify which:

27. Have you read some text about OD (including blog posts)? *

Check only one category

Yes, between 1- 3
Between 4-7
More than 8
No

28. Have you assisted to some talk/conference about OD? *

Check only one category

Yes, between 1- 3
Between 4-7
More than 8
No

29. Have you seen some video either listened some podcast about OD? *

Check only one category

Yes, between 1- 3
Between 4-7
More than 8

No

30. When did you first hear about the OD?*

Check only one category

The last year
In the last 2-5 years
Does further of 5 years

31. How did you met the OD frame? Through: *

Check only one category

Relative/friend
Professional
Association
Social networks
Public assistance
Other

32. If you have answered Other in the ask former specify which

____

33. What do you remember that struck you most about the OD when you first met it?*

Mark one or more

- Its results in psychosis
- To work in the community
- To work with the family/environment/social network
- To organise the whole MH system in the region of origin
- To work in the households
- To work with several professionals together in the same meetings
- To work on demand
- Treatment decisions to be made by consensus.
- Its non-psychiatrising language
- Other: ______________________________

33. If you have answered Other in the ask former specify which:

____

34. Which profile is most relevant to your current situation?*

Check only one category

A. People with own experiences in mental health and OD
B. Peers/ Network
C. Mental health professionals
D. Public mental health system managers/associations
E. University professors/students
F. University students

35. Finally, we welcome your thoughts, ideas, comments, observations and opinions on the DA in Spain:

____
____
____
____
____

We would appreciate that could spread the survey to who think that you may be of interest.

THANK YOU VERY MUCH FOR YOUR COLLABORATION

Please note that the questionnaire was presented using the interface offered by the google forms service and here is a transcription of it for ease of consultation.
